# Role of Helicity on the Anticancer Mechanism of Action of Cationic-Helical Peptides

**DOI:** 10.3390/ijms13066849

**Published:** 2012-06-05

**Authors:** Yi-Bing Huang, Li-Yan He, Hong-Yu Jiang, Yu-Xin Chen

**Affiliations:** 1 Key Laboratory for Molecular Enzymology and Engineering of the Ministry of Education, Jilin University, Changchun 130012, China; E-Mails: huangyibing@jlu.edu.cn (Y.-B.H.); heliyan415@163.com (L.-Y.H.); 2 The First Hospital, Jilin University, Changchun 130021, China

**Keywords:** anticancer peptides, helicity, hydrophobicity, mechanism of action, specificity

## Abstract

In the present study, the 26-residue amphipathic α-helical peptide A12L/A20L (Ac-KWKSFLKTFKSLKKTVLHTLLKAISS-amide) with strong anticancer activity and specificity was used as the framework to study the effects of helicity of α-helical anticancer peptides on biological activities. Helicity was systematically modulated by introducing d-amino acids to replace the original l-amino acids on the non-polar face or the polar face of the helix. Peptide helicity was measured by circular dichroism spectroscopy and was demonstrated to correlate with peptide hydrophobicity and the number of d-amino acid substitutions. Biological studies showed that strong hemolytic activity of peptides generally correlated with high hydrophobicity and helicity. Lower helicity caused the decrease of anti-HeLa activity of peptides. By introducing d-amino acids to replace the original l-amino acids on the non-polar face or the polar face of the helix, we improved the therapeutic index of A12L/A20L against HeLa cells by 9-fold and 22-fold, respectively. These results show that the helicity of anticancer peptides plays a crucial role for biological activities. This specific rational approach of peptide design could be a powerful method to improve the specificity of anticancer peptides as promising therapeutics in clinical practices.

## 1. Introduction

Cancer has become the most malignant diseases threatening human health and life [1,2]. The widely-used traditional treatments against cancer such as chemotherapy or radioactive treatments generally exhibit less specificity, for example, the process that the chemotherapeutic agents kill cancer cells is often associated with deleterious side-effects, including damages to healthy cells and tissues, and lead to chemical resistance whereby many adaptation mutations of cancer cells [3,4]. Therefore, the development of a new class of anticancer agents has become critical.

Nowadays, more and more cationic peptides whether from natural or synthetic sources have been reported to show anticancer activity with various characteristics, like the ability to kill target cells rapidly, the broad spectrum of activity, and the specificity for cancer cells [5]. The cationic α-helical anticancer peptides was a large class of the anticancer peptides, termed as α-ACPs, which often rich in Lys and/or Arg amino acids resulting in the net positive charge of the peptide molecule. Although the mechanism of action of α-ACPs killing the cancer cells has not been clarified yet, it is believed that α-ACPs interact with cancer cell membrane and lead to the cell lysis and cell death [3,5]. There were two general effects of α-ACPs against cancer cells were suggested: cytoplasmic membrane disruption via micellization or pore formation, and induction of apoptosis [6].

For anticancer peptides, the major barrier to use anticancer peptides in clinical practices is the toxicity or ability to lyse eukaryotic cells. Compare to the normal cells, the membrane of the cancer cells have several different properties, (a) the membrane of cancer cells containing with negatively charged phospatidylserine (PS) [7] and *O*-glycosylated mucins [8], which could be attracted to cationic α-ACPs through electrostatic interactions; (b) the membrane fluidity of cancer cell is greater than normal cells, which may enhance the lytic activity of α-ACPs by facilitating membrane destabilization [9]; (c) cell surface areas of cancer cells were greater than normal cells, which is based on the relatively higher number of microvilli on tumorigenic cell that may allow cancer cells to bind increased numbers of α-ACPs [10]. The composition difference of cell membranes between cancer cells and normal cells provides a target of designing and developing new anticancer peptide therapeutics with high specificity.

In the previous study, we have demonstrated that the α-helical cationic anticancer peptides killed various cancer cells with a fast necrotic mechanism causing cell membrane lysis, and peptide hydrophobicity played a crucial role during the action [11]. In this study, the helicity was selected to study the relationship of peptide biophysical properties and the mechanism of action against cancer cells. In order to alter the peptide helicity, a series of d- and l-diastereomeric peptides were designed by introducing d-amino acids to replace the original l-amino acids of α-ACPs. By comparing the helicity and the biological activities of peptides, we illustrated the role of helicity of α-ACPs during the mechanism of action against cancer cells and optimized the anticancer activity of peptide analogs as potential anticancer therapeutics.

## 2. Results

### 2.1. Peptide Design

**Figure 1:**
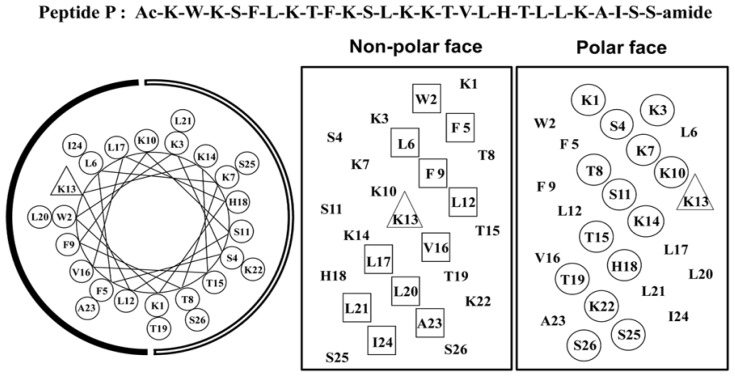
Representation of the parent peptide A12L/A20L as helical net showing the polar/hydrophilic face (circled residues) and non-polar/hydrophobic face (boxed residues) and helical wheel, the lysine residue at position 13 on the non-polar face of the sequence is denoted by a triangle. The hydrophilic face is indicated as an open arc, the hydrophobic face is shown as a solid arc in the helical wheel, Ac denotes *N*^α^-acetyl, and amide denotes *C*^α^-amide. One-letter codes are used for the amino acid residues.

Previously, we have systematically studied the effects of peptide hydrophobicity on the mechanism of action of α-helical cationic anticancer peptides and demonstrated peptides killed various cancer cells with a fast necrotic mechanism causing cell membrane lysis and hydrophobicity plays a crucial role during the action [11]. Among all the anticancer peptides, peptide A12L/A20L showed the strongest anti-HeLa activity; in addition, peptides with greater hydrophobicity than A12L/A20L exhibited more cytotoxicity against normal cells [11]. In this study, in order to illustrate the relationships of hydrophobicity and helicity with the biological activity of amphipathic α-helical anticancer peptides, anticancer peptide A12L/A20L was used as the parent peptide (referred to as peptide P in this study, Figure 1). Helicity was systematically reduced in various degrees by replacing l-lysine residues with d-lysine residues on the polar face of peptide P and l-leucine residues with d-leucine residues on the non-polar face, respectively, since previous studies have showed that d-amino acids exhibit helix-destabilizing properties in an α-helical structure without changing the intrinsic amino acid hydrophobicity [12,13]. The peptide sequences are shown in Table 1 and named according to the substitution sites, for example, K7_D_ means using d-lysine to replace l-lysine at the position 7 on the polar face of peptide P and L6_D_ means using d-leucine to replace l-leucine at the position 6 on the non-polar face of peptide P.

### 2.2. Peptide Secondary Structure

**Figure 2:**
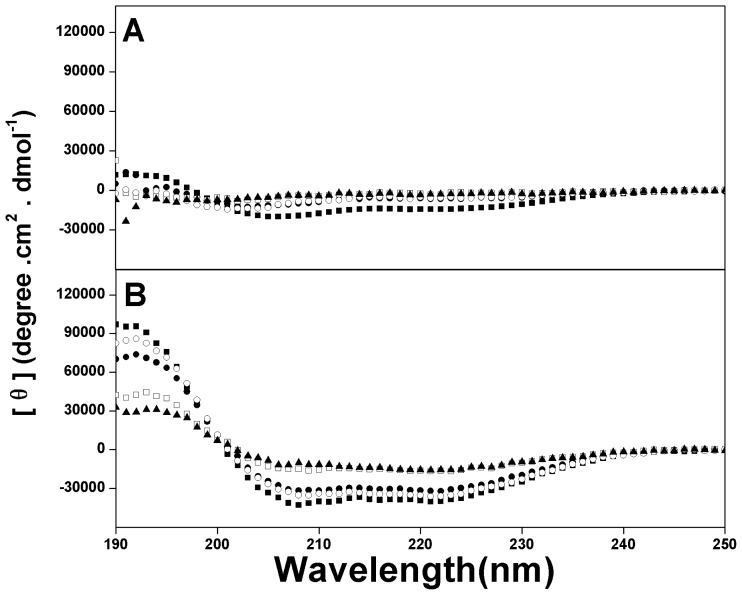
Circular dichroism (CD) spectra of peptide P and its analogs in KP buffer (50 mM KH_2_PO_4_,/K_2_HPO_4_, 100 mM KCl, pH 7.4) (**A**) and in the presence of KP buffer and TFE (1:1 v/v) (**B**) at pH 7.4, 25 °C. Symbols used are ■ for peptide P; ● for peptide K14_D_/K22_D_; □ for peptide K1_D_/K3_D_/K7_D_/K10_D_/K14_D_/K22_D_; ○ for peptide L12_D_/L20_D_ and △ for peptide L6_D_/L12_D_/L17_D_/L20_D_/L21_D_.

To determine the helix-destabilizing effect of d-amino acids in the anticancer peptide P, CD spectra of two sets of peptide analogs with d-lysine substitutions on the polar face and d-leucine substitutions on the non-polar face were measured. Figure 2 shows the CD spectra of the example anticancer peptide analogs under benign conditions and in the presence of 50% TFE to mimic the hydrophobic environment of the membrane. All peptides exhibited negligible helical structure in KP buffer. In contrast, all peptides showed the typical α-helical structure with double minima at 208 nm and 222 nm in the presence of 50% TFE. The molar ellipticity values at different environments and the relative helicity of peptide analogs are shown in Table 2. It is clear to see that peptide helicity was strongly influenced by the number and position of the substituted d-amino acids. In general, helical content of peptide P in the hydrophobic environment was gradually decreased with the increasing number of d-amino acids substitutions both on the polar face and on the non-polar face. For instance, in the presence of 50% TFE, peptide P showed the strongest α-helical structure and the peptide analogs with six d-lysine substitutions on the polar face (K1_D_/K3_D_/K7_D_/K10_D_/K14_D_/K22_D_) and with five d-leucine substitutions on the non-polar face (L6_D_/L12_D_/L17_D_/L20_D_/L21_D_) exhibited only little α-helical contents in the hydrophobic environment (relative helicity of 49% and 33%, respectively) (Table 2).

It is interesting to see that the relative helicity values of peptide K7_D_/K14_D_ (71.7%) and L6_D_/L12_D_ (73.1%) were lower than those of the peptide K14_D_/K22_D_ (81%) and L12_D_/L20_D_ (75.7%) respectively, indicating that *N*-terminal amino acids in the peptide P sequence may be more important to stabilize the helical structure. Furthermore, the d-amino acids substitutions on the polar face of the helix had dramatically stronger effects on the α-helical structure in the hydrophobic environment than the corresponding substitutions on the non-polar face. For example, relative helicity values of single d-amino acid substituted peptides K7_D_ (77.8%), K14_D_ (88.7%) and K22_D_ (85.5%) were less than those of L6_D_ (99.6%), L12_D_ (95.1%) and L20_D_ (92.9%), these results demonstrated that the l-lysine on the polar face had more important role to sustain the helical structure than l-leucine on the non-polar face.

### 2.3. Peptide Hydrophobicity

Peptide relative hydrophobicity was measured by RP-HPLC retention time, since RP-HPLC is highly sensitive to the change of peptide secondary structure during the elution and α-helical peptides bind to the stationary phase of RP-HPLC with the preferred binding domain [13,14]. In general, peptide hydrophobicity was changed in two ways, the difference of intrinsic hydrophobicity of side-chains of substituting amino acids [15] and the alteration of the number of the *i*→*i+3* and *i*→*i+4* hydrophobic interactions of large hydrophobes which affects the continuity of the hydrophobic face of the peptide [16]. However, in this study, the change of peptide hydrophobicity was mainly due to the destabilizing ability of d-amino acids in the α-helical structure and the change of the continuity of the hydrophobic/hydrophilic face of the helix with different d-amino acid substitutions, since d- and l-amino acid enantiomers have the same intrinsic hydrophobicity. In Table 2, the hydrophobicity of peptides (as expressed by RP-HPLC retention time *t_R_*) decreased gradually with the increasing number of d-amino acid substitutions both on the polar face and on the non-polar face of peptide analogs while *t_R_* ranging from 46.9 to 34.6 min (from peptide P to peptide K1_D_/K3_D_/K7_D_/K10_D_/K14_D_/K22_D_) and 46.9 to 34.8 min (from peptide P to peptide L6_D_/L12_D_/L17_D_/L20 _D_/L21_D_), respectively. These results were consistent with the aforementioned orders of helicity on both the polar and non-polar faces.

### 2.4. Anticancer Activity

According to the previous study [11], HeLa cell line was chosen as the target cancer cell line for this study due to its sensitivity to different peptide analogs and the great viability during cell culture process. Peptides have been proved showing no different anti-HeLa activity after the incubation of 1 h or 36 h [11], thus, in this study the anticancer activity of the peptides was determined after incubating with HeLa cells for 1 h and results are shown in Table 3.

From Table 3, it is clear to see that the anticancer activities of peptides correlated to the number of d-leucine substitutions on the non-polar face of the peptides, that is, the more d-amino acid substitutions on the non-polar face, the less anticancer activity the peptides exhibited. In contrast, for the d-lysine substitutions on the polar face, the overall trend was not as clear as that on the non-polar face, indicating the importance of the non-polar face of peptides during the mechanism of action against cancer cells. The single and double d-amino acid substituted peptides (on both the polar and the non-polar faces) exhibited close anticancer activity on IC_50_ values compare to peptide P (1.71 μmol/L), ranging from 1.29 μmol/L to 2.06 μmol/L on the polar face (K7_D_ to K7_D_/K14_D_) and from 2.23 μmol/L to 3.14 μmol/L on the non-polar face (L6_D_ to L12_D_/L20_D_) (Table 3). However, along with the further increase of d-amino acid substitutions, peptide analogs exhibited significant lower anticancer activities compared to peptide P. It is worthy to note that among the peptide analogs with substitutions on the polar face, all three single (K7_D_, K14_D_ and K22_D_) and one double (K14_D_/K22_D_) d-amino acid substituted peptides exhibited stronger anticancer activity than peptide P, showing that maintaining the complete hydrophobic face while modulating the peptide helicity on the polar face may optimize peptide anticancer activity.

### 2.5. Hemolytic Activity

The minimal hemolytic concentration (MHC) of the peptide analogs against human erythrocytes was determined as a major measurement of peptide toxicity toward normal cells (Table 3). Compare to the peptide P (MHC = 5.2 μmol/L), the hemolytic activity of peptide analogs was significantly improved up to no detectable hemolysis at the concentration of 325.2 μmol/L by introducing d-amino acids on both the non-polar face and the polar face of the helix. In general, peptide hemolytic activity was correlated with the number of d-amino acid substitutions on both the polar face and the non-polar face of peptides, *i.e.*, the more d-amino acid substitutions, the weaker the hemolytic activity of the peptides was (Table 3).

### 2.6. Peptide Specificity (Therapeutic Index)

The therapeutic index is calculated by the ratio of MHC (hemolytic activity) and IC_50_ (anticancer activity) and used to represent the specificity of potential reagents, thus, larger values in therapeutic index indicate greater anticancer specificity [11,12]. In Table 3, compare to the parent peptide P, the therapeutic indices of peptides against HeLa are generally in a first-increase-then-drop trend with the increasing number of d-amino acid substitutions both on the polar face and on the non-polar face. For the peptides with substitutions on the polar face, the therapeutic index values range from 3.04 to 67.89. Peptide K7_D_/K10_D_/K14_D_/K22_D_ exhibited the highest therapeutic index value of 67.89, representing that the anti-HeLa activity of peptide K7_D_/K10_D_/K14_D_/K22_D_ was 67.89-fold greater than its toxicity against human red blood cells, which is a 22.3-fold increase on specificity compared to peptide P. In contrast, for the peptides with substitutions on the non-polar face, the therapeutic index values range from 4.67 to 50.5 and peptide L6_D_/L12_D_/L17_D_/L20_D_/L21_D_ showed the best specificity against HeLa cells. However, due to the high anticancer activity, peptide L12_D_/L20_D_ exhibited more valuable specificity against cancer cells than peptide L6_D_/L12_D_/L17_D_/L20_D_/L21_D_ with the therapeutic index value 25.89, which is an 8.5-fold increase on specificity compared to peptide P.

## 3. Discussion

In the previous study, we have demonstrated that the hydrophobicity of peptides plays a crucial role in the mechanism of action against cancer cells and the peptides with greater hydrophobicity showed stronger anticancer activity with a necrotic-like membrane disruption mechanism [11]. Shai *et al.* reported a group of short model diastereomeic peptides composed of varying ratios of leucine and lysine and one third of their sequence composed of d-amino acids could lose their cytotoxic effect on normal mammalian cells but preserve biological activity [17–19], in addition, their stability to enzymatic degradation by serum components is an excellent property for anticancer application. In this study, peptide P with strong anticancer activity was used as the parent peptide and the helicity was systematically reduced to different degrees by replacing l-amino acid residues with d-enantiomeric amino acids on the polar face or the non-polar face.

**Figure 3:**
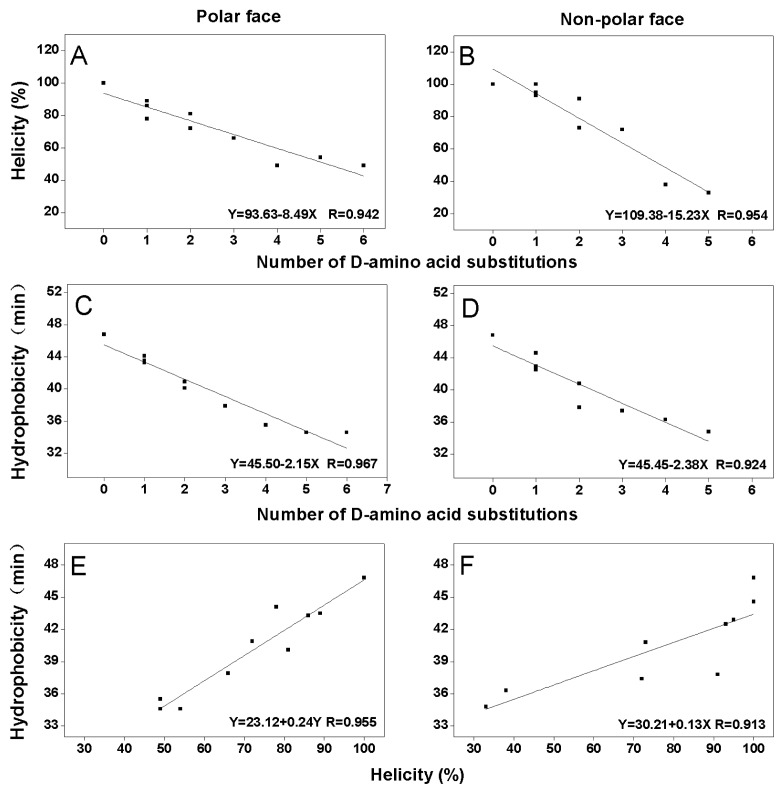
Relationships of helicity, hydrophobicity and the number of d-amino acid substitutions on the polar or the non-polar face of peptide analogs. The experimental data are from Table 2. The least square fit analysis results showed correlations of helicity and number of d-amino acid substitutions with *R* = 0.942 on the polar face (**A**) and *R* = 0.954 on the non-polar face (**B**), correlations of hydrophobicity and number of d-amino acid substitutions with *R* = 0.967 on the polar face (**C**) and *R* = 0.924 on the non-polar face (**D**), and correlations of hydrophobicity and helicity with *R* = 0.955 on the polar face (**E**) and *R* = 0.913 on the non-polar face (**F**).

As shown in Figure 3, peptide helicity in 50% TFE (a mimic of the hydrophobic environment of biomembrane) and hydrophobicity are linearly correlated with the number of d-amino acid substitutions both on the polar face (*R* values of 0.942 and 0.967, respectively) (Figure 3A,C) and on the non-polar face (*R* values of 0.954 and 0.924, respectively) (Figure 3B,D). This can be attributed to the fact that peptides with stronger helicity usually exhibit more complete non-polar face or polar face, thus have higher relative hydrophobicity; whilst, d-amino acid substitutions disrupted α-helical structure of peptides, broke the continuity of the non-polar face or the polar face and reduced the hydrophobicity of peptides [12,13]. At the same times, From Figure 3E,F, it is clear that the hydrophobicity and the helicity in 50% TFE of the peptides with d-amino acid substitutions on the polar face or the non-polar face showed linear correlation with *R* values of 0.955 and 0.913, respectively. These results are consistent with the linear relationships of hydrophobicity and helicity of amphipathic helical anticancer peptides in the previous studies [11,12].

**Figure 4:**
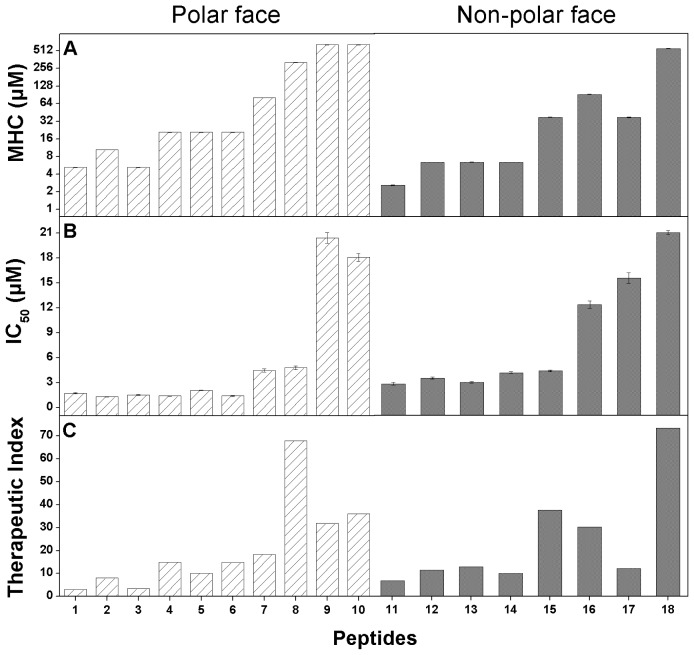
Histogram illustration of relationships of peptide helicity and hemolytic activity (**A**), anticancer activity (**B**) and specificity (**C**). The experimental data are from Table 3. Hatched columns denote the peptides with d-amino acid substitutions on the polar face and solid columns denote the peptides with d-amino acid substitutions on the non-polar face. The numbers on the X-axis denote the corresponding peptide analogs in Table 1.

In this study, the anticancer activity and hemolytic activity of the peptides also correlated with the peptide helicity and the number of d-amino acid substitutions both on the polar face and on the non-polar face (Table 3). However, the helicity showed different effects against cancer cells and normal cells when measured therapeutic index (Table 3, Figure 4). When the number of d-amino acid substitutions on both the polar face and the non-polar face of peptides is less than 3, the anticancer activity of peptide analogs were strong and similar among these peptides, whilst values of the hemolytic activity were showed in a gradually increasing trend, thus peptide specificity were improved. In contrast, when the number of d-amino acid substitutions on both the polar face and the non-polar face of peptides is more than 3, the values of IC_50_ of most peptide analogs increased dramatically (less anticancer activity) with the hemolytic activity improved, resulting the decrease of specificity (Table 3, Figure 4). According to the previous studies, peptides killed cancer cells with a fast necrotic mechanism causing cell membrane lysis as described in the “membrane discrimination mechanism” [11,20,21]. Hydrophobicity plays an important role for peptide penetrating deep into the hydrophobic core of the cell membrane. Helicity showed the similar effect to hydrophobicity during the mechanism of action of anticancer peptides. Stronger helicity usually means the more complete non-polar face of the helix, which is correlated with higher apparent hydrophobicity of peptide molecules when interacting with biomembrane. We believe that hydrophobicity and helicity are key parameters for the mechanism of action of α-helical anticancer peptides. Moreover, in this study, amino acid residues on the polar and the non-polar face of the helix seem to have different effects on peptide biological activity. Amino acids on the polar face may be more sensitive to the cytotoxicity of peptides against normal cells; in contrast, amino acids on the non-polar face are necessary to maintain the anticancer activity. Hence, peptide specificity can be improved by the modulation of suitable d-amino acid on the polar face or the non-polar face of helix.

In summary, this study shows the important role of helicity in the mechanism of action of the α-helical anticancer peptides and the relationships between helicity and hydrophobicity. Utilizing d-amino acid substitution approach, we can modulate peptide helicity, increase anti-HeLa activity and reduce cytotoxicity against normal cells, thus improve peptide specificity. The number of d-amino acid substitutions was correlated with the decrease of peptide helicity and hydrophobicity. This *de novo* design approach proves its value of obtaining new anticancer peptides with promising potentials in clinical practice.

## 4. Experimental Section

### 4.1. Materials

Rink amide 4-methylbenzhydrylamine resin (MBHA resin) (0.8 mmol/g), all of the *N*-α-Fmoc protected amino acids and coupling reagents for peptide synthesis, trifluoroacetic acid (TFA), 2,2,2-trifluoroethanol (TFE) were purchased from GL Biochem (Shanghai, China). Acetonitrile (HPLC grade) was obtained from Fisher Scientific Worldwide Co. (Shanghai, China). 3-(4,5-dimethylthiazol-2-yl)-2,5-diphenyltetrazolium bromide (MTT) was purchased from Sigma (St. Louis, MO, USA). Others analytical grade were purchased from JinXin Chemicals (Changchun, China).

### 4.2. Cell Line and Cell Culture

Human cervix carcinoma cells (HeLa) was obtained from the American Type Culture Collection (ATCC, Manassas, VA, USA) in 2011. In this study, cells were grown at 37 °C in Dulbecco’s modified eagle medium (DMEM) containing 100 U/mL penicillin, 100 μg/mL streptomycin and supplemented with 10% fetal bovine serum (Invitrogen Co., Grand Island, NY, USA).

### 4.3. Peptide Synthesis and Purification

Peptide synthesis was carried out by solid phase peptide synthesis using Fmoc (9-fluorenyl-methoxycar-bonyl) chemistry and Rink amide 4-methylbenzhydrylamine resin (MBHA resin; 0.8 mmol/g), as described previously [12,22]. The crude peptides were purified by preparative Shimadzu LC-6A high-performance liquid chromatography (HPLC), using a Zorbax 300 SB-C_8_ column (250 × 9.4-mm ID, 6.5-μm particle size, 300 Å pore size; Agilent Technologies, Santa Clara, CA, USA) with a linear AB gradient (0.1% acetonitrile/min) at a flow rate of 2 mL/min, while eluent A was 0.1% aqueous trifluoroacetic acid (TFA) in water, and eluent B was 0.1% TFA in acetonitrile. The peptides were further characterized by mass spectrometry and amino acid analysis.

### 4.4. Analytical RP-HPLC of Peptides

Peptide samples were analyzed on a Shimadzu LC-20A HPLC using a Zorbax 300 SB-C_8_ column (150×4.6-mm ID, 5-μm particle size, 300 Å pore size) from Agilent Technologies, Santa Clara, CA, USA) with a linear AB gradient (1% acetonitrile/min) and a flow rate of 1 mL/min, in which eluent A was 0.1% aqueous TFA and eluent B was 0.1% TFA in acetonitrile.

### 4.5. Circular Dichroism Spectroscopy

Circular dichroism (CD) spectra were acquired with a 0.02-cm path length quartz cuvette on a Jasco J-810 spectropolarimeter (Jasco) at 25 °C according to previously described [22]. The concentration of 75 μmol/L peptides was measured in benign conditions (50 mM KH_2_PO_4_/K_2_HPO_4_, 100 mM KCl, PH 7, referred to as KP buffer) or KP buffer with 50% TFE. The mean residue molar ellipticities were calculated by the equation [θ] = θ/10lcMn [12]. The relative helicity of the peptides were determined using the values of mean residue molar ellipticities of the peptide analogs at 222 nm.

### 4.6. Measurement of Anticancer Activity

Human cervix carcinoma cells (HeLa) were grown at 37 °C in Dulbecco’s modified Eagle’s medium (DMEM) containing 100 U/mL penicillin, 100 mg/mL streptomycin and supplemented with 10% FBS (Invitrogen Co.). The MTT assay has been used to test cytotoxicity of reagents and cell viability. Cells were seeded in 96-well plates and incubated with serially 2-fold diluted concentrations of different peptides (0.6–86 μmol/L) for 1 h at 37 °C. As a negative control, cells were cultured without addition of the peptides. Thereafter, 200 μL of 5 mg/mL MTT solution in PBS was added to the cells and treated for 4 h at 37 °C. The formazan crystals were dissolved by adding 150 μL dimethyl sulfoxide (DMSO) just before spectrometric determination. The absorbance was determined at 490 nm. The results were expressed as IC_50_, representing the concentration at which cell viability was reduced by 50%. The cytotoxicity assays were repeated in triplicates.

### 4.7. Measurement of Hemolytic Activity

Peptide samples were serially diluted by PBS in 96-well plates (round bottom) to give a volume of 70 μL sample solution in each well. Human erythrocytes anticoagulated by EDTAK were collected by centrifugation (1000 rpm) for 5 min, and washed twice by PBS, then diluted to a concentration of 2% in PBS. 70 μL of 2% erythrocytes were added to each well to give a final concentration of 1% human erythrocytes in each well and plates were incubated at 37 °C for 1 h. The plates were then centrifuged for 10 min at 2000 rpm and supernatant (90 μL) was transferred to a 96-well plate (flat bottom). The release of hemoglobin was determined by measuring the absorbance of the supernatant at 578 nm. The hemolytic activity was determined as the minimal peptide concentration that caused hemolysis (minimal hemolytic concentration, MHC). Erythrocytes in PBS and distilled water were used as control of 0% and 100% hemolysis, respectively.

## Figures and Tables

**Table 1:** Design and sequence of α-helical antimicrobial peptides.

Group	No.	Peptide	Amino Acid Sequence^a^
Parent	1	P	Ac-K-W-K-S-F-L-K-T-F-K-S-L-K-K-T-V-L-H-T-L-L-K-A-I-S-S-amide

Polar face group	2	K7_D_	Ac-K-W-K-S-F-L-***K***-T-F-K-S-L-K-K-T-V-L-H-T-L-L-K-A-I-S-S-amide
	3	K14_D_	Ac-K-W-K-S-F-L-K-T-F-K-S-L-K-***K***-T-V-L-H-T-L-L-K-A-I-S-S-amide
	4	K22_D_	Ac-K-W-K-S-F-L-K-T-F-K-S-L-K-K-T-V-L-H-T-L-L-***K***-A-I-S-S-amide
	5	K7_D_/K14_D_	Ac-K-W-K-S-F-L-***K***-T-F-K-S-L-K-***K***-T-V-L-H-T-L-L-K-A-I-S-S-amide
	6	K14_D_/K22_D_	Ac-K-W-K-S-F-L-K-T-F-K-S-L-K-***K***-T-V-L-H-T-L-L-***K***-A-I-S-S-amide
	7	K7_D_/K14_D_/K22_D_	Ac-K-W-K-S-F-L-***K***-T-F-K-S-L-K-***K***-T-V-L-H-T-L-L-***K***-A-I-S-S-amide
	8	K7_D_/K10_D_/K14_D_/K22_D_	Ac-K-W-K-S-F-L-***K***-T-F-***K***-S-L-K-***K***-T-V-L-H-T-L-L-***K***-A-I-S-S-amide
	9	K3_D_/K7_D_/K10_D_/K14_D_/K22_D_	Ac-K-W-***K***-S-F-L-***K***-T-F-***K***-S-L-K-***K***-T-V-L-H-T-L-L-***K***-A-I-S-S-amide
	10	K1_D_/K3_D_/K7_D_/K10_D_/K14_D_/K22_D_	Ac-***K***-W-***K***-S-F-L-***K***-T-F-***K***-S-L-K-***K***-T-V-L-H-T-L-L-***K***-A-I-S-S-amide

Non-polar face group	11	L6_D_	Ac-K-W-K-S-F-***L***-K-T-F-K-S-L-K-K-T-V-L-H-T-L-L-K-A-I-S-S-amide
	12	L12_D_	Ac-K-W-K-S-F-L-K-T-F-K-S-***L***-K-K-T-V-L-H-T-L-L-K-A-I-S-S-amide
	13	L20_D_	Ac-K-W-K-S-F-L-K-T-F-K-S-L-K-K-T-V-L-H-T-***L***-L-K-A-I-S-S-amide
	14	L6_D_/L12_D_	Ac-K-W-K-S-F-***L***-K-T-F-K-S-***L***-K-K-T-V-L-H-T-L-L-K-A-I-S-S-amide
	15	L12_D_/L20_D_	Ac-K-W-K-S-F-L-K-T-F-K-S-***L***-K-K-T-V-L-H-T-***L***-L-K-A-I-S-S-amide
	16	L6_D_/L12_D_/L20_D_	Ac-K-W-K-S-F-***L***-K-T-F-K-S-***L***-K-K-T-V-L-H-T-***L***-L-K-A-I-S-S-amide
	17	L6_D_/L12_D_/L17_D_/L20_D_	Ac-K-W-K-S-F-***L***-K-T-F-K-S-***L***-K-K-T-V-***L***-H-T-***L***-L-K-A-I-S-S-amide
	18	L6_D_/L12_D_/L17_D_/L20_D_/L21_D_	Ac-K-W-K-S-F-***L***-K-T-F-K-S-***L***-K-K-T-V-***L***-H-T-***L***-***L***-K-A-I-S-S-amide

a One-letter codes are used for the amino acid residues; the bold italic letters denote the substituting D-amino acids of the peptide P, all other amino acids are L-amino acids.

**Table 2:** Biophysical data of the peptide analogs.s

Peptides a	*t*_R_ (min) b 25 °C	Benign c		50% TFE d	

		[θ]_222_	% helix e	[θ]_222_	% helix e
P	46.9	−14550	36.66	−39700	100.00
K7_D_	44.1	−6050	15.22	−30900	77.77
K14_D_	43.5	−15550	39.16	−35250	88.72
K22_D_	43.3	−8400	21.14	−33950	85.48
K7_D_/K14 _D_	40.9	−8750	22.02	−28450	71.65
K14_D_/K22_D_	40.1	−6350	15.95	−32150	81.00
K7_D_/K14_D_/K22_D_	37.9	−5000	12.59	−26350	66.33
K7_D_/K10_D_/K14_D_/K22_D_	35.5	−7350	22.48	−19400	48.84
K3_D_/K7_D_/K10_D_/K14_D_/K22_D_	34.6	−4750	14.59	−21400	53.99
K1_D_/K3_D_/K7_D_/K10_D_/K14_D_/K22_D_	34.6	−5800	17.74	−19300	48.61
L6_D_	44.6	−8500	21.45	−39550	99.61
L12_D_	42.9	−8350	21.01	−37750	95.08
L20_D_	42.5	−6900	17.42	−36900	92.90
L6_D_/L12_D_	40.8	−6800	17.14	−29050	73.13
L12_D_/L20_D_	37.8	−5550	13.93	−30050	75.69
L6_D_/L12_D_/L20_D_	37.4	−4950	12.45	−28450	71.66
L6_D_/L12_D_/L17_D_/L20_D_	36.3	−4050	12.46	−15050	37.88
L6_D_/L12_D_/L17_D_/L20_D_/L21_D_	34.8	−3600	10.97	−13150	33.20

a Peptides are ordered by relative hydrophobicity;

b *t*_R_ (min) denotes the retention time at 25 °C by RP-HPLC;

c The mean residue molar ellipticities, [θ]_222_ (degree·cm^2^·dmol^−1^) at wavelength 222 nm were measured at 25 °C in KP buffer (100 mM KCl, 50 mM PO_4_, pH 7.0);

d The mean residue molar ellipticities, [θ]_222_ (degree·cm^2^·dmol^−1^) at wavelength 222 nm were measured at 25 °C in KP buffer with 50% TFE;

e The helical content (in percentage) of a peptide relative to the molar ellipticity value of peptide P in 50% TFE.

**Table 3:** Biological data of peptide analogs.

Peptides a	MHC b (μmol/L)	IC_50_ c (μmol/L)	Therapeutic Index d	Fold e
P	5.20 ± 0.02	1.71 ± 0.07	3.04	1.0
K7_D_	10.4 ± 0.04	1.29 ± 0.03	8.07	2.7
K14_D_	5.20 ± 0.02	1.52 ± 0.05	3.42	1.1
K22_D_	20.81 ± 0.10	1.39 ± 0.02	14.97	4.9
K7_D_/K14_D_	20.81 ± 0.15	2.06 ± 0.01	10.10	3.3
K14_D_/K22_D_	20.81 ± 0.06	1.40 ± 0.09	14.86	4.9
K7_D_/K14_D_/K22_D_	81.31 ± 0.17	4.45 ± 0.19	18.27	6.0
K7_D_/K10_D_/K14_D_/K22_D_	325.20 ± 0.82	4.79 ± 0.23	67.89	22.3
K3_D_/K7_D_/K10_D_/K14_D_/K22_D_	>325.20	20.41 ± 0.64	31.87	10.5
K1_D_/K3_D_/K7_D_/K10_D_/K14_D_/K22_D_	>325.20	18.07 ± 0.48	35.99	11.8
L6_D_	10.40 ± 0.08	2.23 ± 0.10	4.67	1.5
L12_D_	20.81 ± 0.03	2.63 ± 0.07	7.91	2.6
L20_D_	20.81 ± 0.13	2.33 ± 0.06	8.93	2.9
L6_D_/L12_D_	20.81 ± 0.10	3.01 ± 0.08	6.91	2.3
L12_D_/L20_D_	81.31 ± .43	3.14 ± 0.06	25.89	8.5
L6_D_/L12_D_/L20_D_	162.61 ± 0.19	7.80 ± 0.26	20.85	6.9
L6_D_/L12_D_/L17_D_/L20_D_	81.31 ± 1.05	9.69 ± 0.38	8.39	2.8
L6_D_/L12_D_/L17_D_/L20_D_/L21_D_	>325.20	12.88 ± 0.15	50.50	16.6

a Peptides are ordered by relative hydrophobicity;

b Hemolytic activity (minimal hemolytic concentration) was determined on human red blood cells after incubating with peptides for 1 h (hRBC); When no hemolytic activity was observed at 325.2 μmol/L, a value of 650.4 μmol/L was used for calculation of the therapeutic index;

c Anticancer activity (IC_50_) represents the concentration of peptides at which cell viability was reduced by 50% in comparison to untreated cells; The MTT assay was repeated in triplicate and IC_50_ value was determined by averaging three repeated experiments;

d Therapeutic index = MHC/IC_50_, Larger values indicate greater anticancer specificity;

e The fold improvement in the therapeutic index was determined as relative to that of parent peptide P.

## References

[1] Edwards B.K., Brown M.L., Wingo P.A., Howe H.L., Ward E., Ries L.A., Schrag D., Jamison P.M., Jemal A., Wu X.C. (2005). Annual report to the nation on the status of cancer, 1975–2002, featuring population-based trends in cancer treatment. J. Natl. Cancer Inst.

[2] Jemal A., Bray F., Center M.M., Ferlay J., Ward E., Forman D. (2011). Global cancer statistics. CA Cancer J. Clin.

[3] Hoskin D.W., Ramamoorthy A. (2008). Studies on anticancer activities of antimicrobial peptides. Biochim. Biophys. Acta.

[4] Eisenhofer G., Siegert G., Kotzerke J., Bornstein S.R., Pacak K. (2008). Current progress and future challenges in the biochemical diagnosis and treatment of pheochromocytomas and paragangliomas. Horm. Metab. Res.

[5] Dennison S.R., Whittaker M., Harris F., Phoenix D.A. (2006). Anticancer alpha-helical peptides and structure/function relationships underpinning their interactions with tumour cell membranes. Curr. Protein Pept. Sci.

[6] Papo N., Shai Y. (2005). Host defense peptides as new weapons in cancer treatment. Cell Mol. Life Sci.

[7] Ran S., Downes A., Thorpe P.E. (2002). Increased exposure of anionic phospholipids on the surface of tumor blood vessels. Cancer Res.

[8] Yoon W.H., Park H.D., Lim K., Hwang B.D. (1996). Effect of *O*-glycosylated mucin on invasion and metastasis of HM7 human colon cancer cells. Biochem. Biophys. Res. Commun.

[9] Sok M., Sentjurc M., Schara M. (1999). Membrane fluidity characteristics of human lung cancer. Cancer Lett.

[10] Chaudhary J., Munshi M. (1995). Scanning electron microscopic analysis of breast aspirates. Cytopathology.

[11] Huang Y.B., Wang X.F., Wang H.Y., Liu Y., Chen Y. (2011). Studies on mechanism of action of anticancer peptides by modulation of hydrophobicity within a defined structural framework. Mol. Cancer Ther.

[12] Chen Y., Mant C.T., Farmer S.W., Hancock R.E., Vasil M.L., Hodges R.S. (2005). Rational design of alpha-helical antimicrobial peptides with enhanced activities and specificity/therapeutic index. J. Biol. Chem.

[13] Chen Y., Mant C.T., Hodges R.S. (2002). Determination of stereochemistry stability coefficients of amino acid side-chains in an amphipathic alpha-helix. J. Pept. Res.

[14] Zhou N.E., Mant C.T., Hodges R.S. (1990). Effect of preferred binding domains on peptide retention behavior in reversed-phase chromatography: Amphipathic alpha-helices. Pept. Res.

[15] Kovacs J.M., Mant C.T., Hodges R.S. (2006). Determination of intrinsic hydrophilicity/hydrophobicity of amino acid side chains in peptides in the absence of nearest-neighbor or conformational effects. Biopolymers.

[16] Chen Y., Guarnieri M.T., Vasil A.I., Vasil M.L., Mant C.T., Hodges R.S. (2007). Role of peptide hydrophobicity in the mechanism of action of alpha-helical antimicrobial peptides. Antimicrob. Agents Chemother.

[17] Oren Z., Hong J., Shai Y. (1997). A repertoire of novel antibacterial diastereomeric peptides with selective cytolytic activity. J. Biol. Chem.

[18] Papo N., Shai Y. (2003). New lytic peptides based on the d,l-amphipathic helix motif preferentially kill tumor cells compared to normal cells. Biochemistry.

[19] Papo N., Shahar M., Eisenbach L., Shai Y. (2003). A novel lytic peptide composed of dl-amino acids selectively kills cancer cells in culture and in mice. J. Biol. Chem.

[20] Shai Y. (1999). Mechanism of the binding, insertion and destabilization of phospholipid bilayer membranes by alpha-helical antimicrobial and cell non-selective membrane-lytic peptides. Biochim. Biophys. Acta.

[21] Chen Y., Vasil A.I., Rehaume L., Mant C.T., Burns J.L., Vasil M.L., Hancock R.E., Hodges R.S. (2006). Comparison of biophysical and biologic properties of alpha-helical enantiomeric antimicrobial peptides. Chem. Biol. Drug Des.

[22] Huang J.F., Xu Y.M., Hao D.M., Huang Y.B., Liu Y., Chen Y.X. (2010). Structure-guided *de novo* design of α-helical antimicrobial peptide with enhanced specificity. Pure Appl. Chem.

